# Fulvestrant and the sequential endocrine cascade for advanced breast cancer

**DOI:** 10.1038/sj.bjc.6601632

**Published:** 2004-03-05

**Authors:** S Johnston

**Affiliations:** 1 Department of Medicine (Breast Unit), The Royal Marsden Hospital NHS Trust & Institute of Cancer Research, Fulham Road, London SW3 6JJ, UK

**Keywords:** breast cancer, endocrine therapy, sequencing, fulvestrant, ‘Faslodex’

## Abstract

Following relapse on endocrine therapy for advanced, hormone receptor-positive breast cancer, it is common for patients to experience responses to alternative endocrine agents. Fulvestrant (‘Faslodex’) is a new type of endocrine treatment – an oestrogen receptor (ER) antagonist with no agonist effects. Fulvestrant downregulates cellular levels of the ER resulting in decreased expression of the progesterone receptor. This unique mode of action means that it is important that fulvestrant is placed optimally within the sequence of endocrine therapies to ensure that patients gain maximum benefit. Fulvestrant has shown efficacy when used after progression on tamoxifen or anastrozole in postmenopausal women with advanced breast cancer. After progression on fulvestrant, subsequent endocrine treatments can produce responses in many patients, demonstrating that fulvestrant does not lead to crossresistance with other endocrine therapies. Responses to fulvestrant have also been observed in patients heavily pretreated with prior endocrine therapy. Fulvestrant is a versatile endocrine agent that may be integrated into the therapeutic sequence prior to, or subsequent to, other hormonal therapies, and represents a valuable additional antioestrogen for the treatment of postmenopausal women with advanced breast cancer.

The efficacy and tolerability advantages associated with the use of endocrine agents in the treatment of hormone receptor-positive advanced breast cancer have been clearly established in many clinical studies. However, despite an initial response, many patients will eventually experience disease progression and require further endocrine treatment options. In patients who respond to endocrine treatments, additional responses to further agents are common (Buzdar and Hortobagyi, 1998; Hortobagyi, 1998). This potential responsiveness to multiple endocrine therapies means that patients may continue to derive clinical benefit while avoiding the marked, and often distressing, adverse side effects associated with chemotherapy. This is a particularly important consideration in a predominantly elderly patient population who may be least able to tolerate severe adverse events. Disease control is also important in this patient group for whom an absolute cure may not be achievable, and instead, prevention of disease progression and the maintenance of quality of life may be more important.

The activity of sequential endocrine therapies is dependent upon them possessing different mechanisms of action. In this way, crossresistance between sequential therapies may be avoided. It is therefore important that, as new endocrine therapies with different mechanisms of action become available, they are integrated effectively into the sequential hormonal regimens to allow patients to derive maximum benefit.

## FULVESTRANT AND THE ENDOCRINE SEQUENCE CASCADE

Fulvestrant (‘Faslodex’) is a new type of endocrine treatment – an oestrogen receptor (ER) antagonist with no agonist effects (Wakeling *et al*, 1991; Robertson *et al*, 2001). Fulvestrant binds to the ER but, due to its steroidal structure and long side-chain, induces a different conformational shape with the receptor to that achieved by the nonsteroidal antioestrogen tamoxifen. As a result of this, fulvestrant prevents ER dimerisation and leads to the rapid degradation of the fulvestrant–ER complex, producing the loss of cellular ER (Borras *et al*, 1996). As a result, fulvestrant (unlike tamoxifen) inhibits ER–DNA binding and produces abrogation of oestrogen-sensitive gene transcription (Dauvois *et al*, 1993).

The unique mode of action of fulvestrant presents a useful addition to the endocrine agents currently available for use in sequential therapeutic regimens. Fulvestrant has been approved in the United States and Brazil for the treatment of hormone receptor-positive metastatic breast cancer in postmenopausal women with disease progression following antioestrogen therapy. An increasing number of studies are demonstrating the versatility of fulvestrant for the treatment of advanced breast cancer (Howell *et al*, 2002; Osborne *et al*, 2002; Perey *et al*, 2002; Steger *et al*, 2003a, 2003b). An understanding and appreciation of these data will be important for determining the optimal placing of fulvestrant in the sequence cascade of hormonal therapy.

### Efficacy post-tamoxifen

The efficacy of fulvestrant has been proven in two phase III trials conducted in postmenopausal patients with hormone-sensitive advanced breast cancer progressing on prior tamoxifen. In both these trials, the efficacy of fulvestrant was comparable to the highly selective, third-generation aromatase inhibitor (AI) anastrozole (‘Arimidex’) (Howell *et al*, 2002; Osborne *et al*, 2002). Fulvestrant is the only antioestrogen acting directly on ER that has demonstrated efficacy post-tamoxifen, illustrating the lack of crossresistance between these two therapies. This is in contrast to the selective oestrogen receptor modulators (SERMs) such as droloxifene, idoxifene, toremifene, and benzothiophene arzoxifene, all of which have shown minimal activity in tamoxifen-resistant disease (Johnston, 2001).

### Efficacy post-AI

Owing to their improved efficacy and tolerability, AIs are increasingly being used in the first-line treatment of breast cancer, in both early and advanced disease (Nabholtz *et al*, 2000; ATAC Trialists' Group, 2002; Mouridsen *et al*, 2003). Preclinical data indicate that exposure to long-term oestrogen deprivation (similar to that caused by AIs) and subsequent development of acquired resistance may be accompanied by adaptive increases in ER gene expression and intercellular signalling, resulting in hypersensitivity to low oestradiol levels (Jeng *et al*, 1998; Shim *et al*, 2000; Chan *et al*, 2002; Martin *et al*, 2003). In this situation, tamoxifen may be perceived as an agonist. As a result, it is important to establish the efficacy of fulvestrant after progression on AIs. *In vitro*, fulvestrant significantly inhibited the expression of genes such as c-*myb* and c-*myc* in cells resistant to long-term oestrogen deprivation (Jeng *et al*, 1998) and may therefore be an appropriate therapeutic option after progression on AIs.

Clinical data so far are limited, but preliminary results from an ongoing phase II study have shown that fulvestrant produced clinical benefit (CB, complete response (CR)+partial response (PR)+stable disease (SD) ⩾24 weeks) in seven out of 17 (41%) patients who had received, and had progressed on, prior treatment with tamoxifen and an AI (Perey *et al*, 2002). These results suggest that in addition to producing responses after prior tamoxifen, disease progression after anastrozole may not preclude subsequent treatment with fulvestrant. Further trials in this setting are now in progress and are discussed later in this paper.

### Efficacy in heavily pretreated patients

Many patients may receive a number of different endocrine therapies as well as chemotherapies during the course of their disease. Preliminary data are becoming available from centres using fulvestrant in Named Patient Programmes involving patients heavily pretreated with endocrine therapies including tamoxifen, anastrozole, letrozole, exemestane, and goserelin. In 67 postmenopausal women with metastatic breast cancer, 64 of whom (96%) had progressed on one, two or three prior endocrine agents for advanced disease, fulvestrant produced CB in 40 patients (60%) overall. A total of six patients (9%) derived a PR. Of these, one had received fulvestrant as first-line therapy for advanced disease, two had received fulvestrant as second-line therapy, and three had received it as third-line therapy. No objective responses were seen in patients receiving fourth-line fulvestrant therapy. This might suggest that fulvestrant produces better responses when given earlier in the treatment sequence (Steger *et al*, 2003a).

Similar results have been obtained in a separate single-centre study. Postmenopausal women with metastatic breast cancer who had been heavily pretreated with prior hormonal therapy (including tamoxifen, AIs, androgens, and high-dose oestrogens) and chemotherapy (including taxanes, capecitabine, doxorubicin, and cisplatin) were treated with fulvestrant; SD ⩾24 weeks was achieved in eight out of 42 (19%) patients (Franco *et al*, 2003).

### Endocrine therapy after progression on fulvestrant

Two studies have provided evidence that the marked reduction in ER expression produced by fulvestrant is not associated with crossresistance to subsequent endocrine therapies (Howell, 2002; Vergote *et al*, 2003). These studies used the retrospective analysis of data derived from questionnaires sent to clinicians who were involved in trials of fulvestrant as first- or second-line therapy (Osborne *et al*, 2002; Robertson *et al*, 2002). This methodology imposes certain limitations on the analyses. However, the information obtained from these studies does provide further evidence with regard to establishing sequencing regimens.

Responses to subsequent endocrine therapy in patients who progressed on fulvestrant or tamoxifen as first-line therapy for advanced disease have been examined in a retrospective analysis (Howell, 2002). The limitations of this analysis are illustrated by the fact that while 170 patients derived CB on fulvestrant, follow-up data on patients who received subsequent endocrine therapy were available for only 35 of these. In these patients, subsequent endocrine therapy produced CB in 20 out of 35 (57%) patients, with AI-based therapy producing CB in 11 out of 22 (50%) patients (Table 1

**Table 1:** Response to subsequent therapy in patients who derived clinical benefit (CB) from fulvestrant

	**Number of patients**				
	**CR**	**PR**	**SD ⩾24 weeks**	**PD**	**Total**
**Patients who derived CB from first-line fulvestrant**					
*Endocrine therapy total*	1	2	17	15	35
Aromatase inhibitors	1	1	9	11	22
Tamoxifen	0	1	7	2	10
Megestrol acetate	0	0	1	0	1
Medroxyprogesterone acetate	0	0	0	2	2
					
**Patients who derived CB from second-line fulvestrant**					
*Endocrine therapy total*	0	4	21	29	54
Aromatase inhibitors	0	3	16	27	46
Megestrol acetate	0	1	5	2	8

Adapted from Howell (2002) with permission of *Breast Cancer Research and Treatment* (Vergote *et al*, 2003). CR=complete response; PR=partial response; SD=stable disease; PD=progressive disease.

). It is interesting to note that in those patients who failed to derive CB from fulvestrant, subsequent endocrine therapy produced a similar number of responses to those seen in patients who did derive CB from fulvestrant (15 out of 35 (43%) and 20 out of 35 (57%), respectively; Table 2

**Table 2:** Response to subsequent therapy in patients who did not derive clinical benefit (CB) from fulvestrant

	**Number of patients**				
	**CR**	**PR**	**SD** ⩾**24 weeks**	**PD**	**Total**
**Patients who did not derive CB from first-line fulvestrant**					
*Endocrine therapy total*	0	3	12	20	35
Aromatase inhibitors	0	0	8	11	19
Tamoxifen	0	3	2	7	12
Megestrol acetate	0	0	1	0	1
Medroxyprogesterone acetate	0	0	1	2	3
					
**Patients who did not derive CB from second-line fulvestrant**					
*Endocrine therapy total*	0	1	17	33	51
Aromatase inhibitors	0	1	15	26	42
Megestrol acetate	0	0	1	5	6
Medroxyprogesterone acetate	0	0	1	2	3

Adapted from Howell (2002) with permission of *Breast Cancer Research and Treatment* (Vergote *et al*, 2003). CR=complete response; PR=partial response; SD=stable disease; PD=progressive disease.

). While the selection of patients and patient numbers included in this analysis are limited by the nature of the data collection, they do indicate that responses may be obtained with AIs and other endocrine therapies after progression on fulvestrant.

In another retrospective analysis, 186 patients in total derived CB on second-line fulvestrant, although questionnaire-based follow-up data were available for only 54 of these patients (Vergote *et al*, 2003). The results showed that treatment with endocrine therapy (predominantly AIs) after second-line fulvestrant produced CB in 25 out of 54 (46%) patients and objective response (OR, CR+PR) in four out of 54 (7%) patients who obtained CB with second-line fulvestrant (Table 1). In patients who failed to derive CB from second-line fulvestrant, further endocrine therapy produced CB in 18 out of 51 (35%) patients and OR in one out of 51 (2%) patients (Table 2). Preliminary analyses demonstrated a median duration of response to subsequent therapy of 383 and 318 days, for patients who did and who did not derive CB from second-line fulvestrant, respectively. Further endocrine therapy after progression on fulvestrant is therefore a viable and effective therapeutic option, with responses seen in patients treated with tamoxifen and megestrol acetate as well as AIs such as anastrozole and letrozole.

## DISCUSSION

It is important to be aware of the sequence versatility of fulvestrant so that it may be effectively and appropriately incorporated into the endocrine sequence cascade. Fulvestrant has demonstrated efficacy in the treatment of postmenopausal women with advanced, hormone-sensitive breast cancer, with data indicating that fulvestrant exhibits this activity in both the post-tamoxifen and postanastrozole setting. More specifically, fulvestrant has been shown to be at least as effective as anastrozole in women with hormone-sensitive disease who have progressed on first-line therapy (mainly tamoxifen) (Howell *et al*, 2002; Osborne *et al*, 2002; Robertson *et al*, 2003), with preliminary data showing promising results after progression on AIs (Perey *et al*, 2002). Fulvestrant has also been used in patients pretreated with several endocrine agents as well as chemotherapy. In one report, an overall CB rate of 60% was obtained, although patients who were treated with fulvestrant earlier in the sequence appeared to obtain better responses than those who received it after progression on three endocrine agents (Steger *et al*, 2003a).

**Figure 1:**
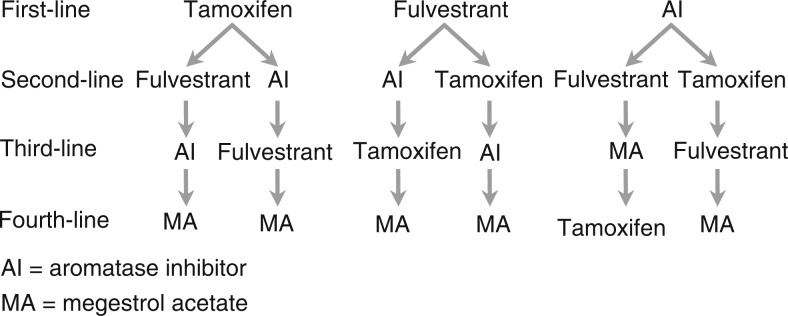
Proposed positions of fulvestrant within the available endocrine therapies for the sequential treatment of postmenopausal women with hormone receptor-positive, advanced disease. Adapted from Carlson (2002) with permission of *Breast Cancer Research and Treatment*.

In patients who undergo disease progression on fulvestrant, the novel mode of action of this new endocrine therapy ensures a lack of crossresistance to other current endocrine agents (Howell, 2002; Vergote *et al*, 2003). Thus, the early use of fulvestrant in the sequence of endocrine therapies may not limit later choices of endocrine therapy. Fulvestrant can, therefore, potentially be integrated into sequential endocrine regimens at a number of positions, including the second-line setting after tamoxifen, or, potentially, after AIs (Figure 1

). Endocrine therapies such as megestrol acetate or the steroidal AI exemestane may then be employed as necessary after progression on fulvestrant. In this way, the potentially most effective and well-tolerated agents are used earlier in the treatment sequence.

To optimise the positioning of fulvestrant in the sequence of endocrine therapies, additional studies will be required to elaborate upon the data so far accrued. New phase II and III clinical trials of fulvestrant in over 3000 patients are either planned or currently in progress (Table 3

**Table 3:** New phase II/III clinical trials of fulvestrant in over 3000 breast cancer patients

**Trial**	**Phase**	**Population**	**Treatments**	**Patients (*n*)**
NCCTG	II	Post-tamoxifen or post-AIs	Fulvestrant 250 mg	89
SAKK	II	Post-tamoxifen or post-AIs	Fulvestrant 250 mg	93
EFECT	III	Post-nonsteroidal AI	Fulvestrant LD 250 mg *vs* exemestane	660
SOFEA	III	Post-nonsteroidal AI	Fulvestrant LD 250 mg±anastrozole *vs* exemestane	750
FACT	III	First-line	Fulvestrant LD 250 mg+anastrozole *vs* anastrozole	558
SWOG 226	III	First-line	Fulvestrant 250 mg+anastrozole *vs* anastrozole	690
0057	II	Neoadjuvant	Fulvestrant 250 mg+anastrozole *vs* anastrozole	120
FAST	II	Neoadjuvant	Fulvestrant HD *vs* tamoxifen	60

NCCTG=North Central Cancer Treatment Group; SAKK=Swiss Group for Clinical Cancer Research; EFECT=Evaluation of Faslodex *vs* Exemestane Clinical Trial; SOFEA=Study Of Faslodex *vs* Exemestane with/without Arimidex; SWOG=Southwest Oncology Group; LD 250 mg=loading-dose schedule of fulvestrant: 500 mg day 0, 250 mg days 14 and 28, fulvestrant 250 mg per monthly thereafter; HD=high-dose schedule of fulvestrant 750 mg 2–3 weeks presurgery.

). These will investigate additional roles for fulvestrant in breast cancer therapy, either following prior nonsteroidal AI treatment or in combination with AIs as first-line therapy. In addition, loading-dose fulvestrant regimens will be tested. Two randomised, controlled trials are comparing the efficacy and tolerability of fulvestrant *vs* exemestane in postmenopausal women progressing after long-term oestrogen deprivation resulting from prior AI therapy. The primary aim of the Study Of Faslodex *vs* Exemestane with/without Arimidex (SOFEA) trial is to compare progression-free survival in patients who have progressed on a nonsteroidal AI, and who are subsequently treated with either fulvestrant plus continued anastrozole, or with fulvestrant alone. Secondary aims include a comparison of fulvestrant *vs* exemestane and an examination of biological markers of response. A further trial, the Evaluation of Faslodex *vs* Exemestane Clinical Trial (EFECT) is currently recruiting patients to assess the efficacy of fulvestrant *vs* exemestane in patients who have progressed on treatment with nonsteroidal AIs. In addition, two trials (FACT and SWOG 226) will compare the efficacy of a combination of fulvestrant plus anastrozole with anastrozole alone in the first-line setting (Table 3). The results of trials such as these will further define endocrine-sequencing strategies, particularly as AIs move forward into the first-line or adjuvant settings.

Currently available data therefore indicate that fulvestrant will be a useful therapeutic option that may extend the opportunity for using endocrine therapies before reliance upon cytotoxic chemotherapy is necessary. Fulvestrant is also a versatile endocrine therapy that may be used at a variety of positions in the sequential use of endocrine therapy for postmenopausal women with advanced, hormone-sensitive breast cancer.
